
JOURNAL INFORMATION
==============================
NLM Title Abbreviation: JBRA Assist Reprod
Journal ID: JBRA Assist Reprod
NLM Journal ID: 101684552
Journal ID: jbra

JBRA Assisted Reproduction

ISSN: 1517-5693
EISSN: 1518-0557
Publisher: Brazilian Society of Assisted Reproduction

ARTICLE INFORMATION
==============================
PMCID: PMC5264384
PMID: 27584612
DOI: 10.5935/1518-0557.20160036
Article version: 1
Subjects: Poster Presentations

Poster Presentations: Abstracts of the 20th Annual Congress of the SBRA, Belo Horizonte/MG 14-17 September 2016

Publication date: 2016 Jul-Sep
Print publication date: 2016 Jul-Sep
Volume: 20
Issue: 3
First page: 168
Last page: 193
License: This is an Open Access article distributed under the terms of the Creative Commons Attribution License, which permits unrestricted use, distribution, and reproduction in any medium, provided the original work is properly cited.
License URL: https://creativecommons.org/licenses/by/4.0/

==============================


P-01. Folliculogenesis progression in ectopic grafted ovarians after vitrification and devitrification followed by exogenous stimulation

Alves Thalys J M 1
Dias Marco T 1
Barreto Filipe P 1
Gonzaga Jhéssica L M 1
Campos-Júnior Paulo H A 1
1 Universidade Federal de São João del Rei/MG, Brazil
First page: 168
Last page: 168

P-02. Efficiency of vitrification-warming blastocyst transfer in regular patients, egg donation program and embryo biopsy cycles: 1.5 years of experience with Ingamed® protocol

Barreto Mariana N 1
Barros Bruna 1
Motta Eduardo L A da 1
Serafini Paulo C 1
Bonetti Tatiana C S 1
Alegretti Jose R 1
1 Huntington Medicina Reprodutiva, Sao Paulo/SP, Brazil
First page: 168
Last page: 168

P-03. Evaluation of biochemical and reproductive parameters of rats submitted to administration of commercial energy drink

Araújo Eduardo S de 1
Schaefer David 1
Nascimento Anna E A do 1
Salvador Rafael A 1
Senn Alfred 2
Til David 1
Frajblat Marcel 3
Amaral Vera Lucia L 1
1 Universidade do Vale do Itajai-UNIVALI, Itajai/SC, Brazil
2 Fondation pour l’Andrologie, la Biologie et l’Endocrinologie de la Reproduction - FABER - Lausanne, Switzerland
3 Universidade Federal do Rio de Janeiro-UFRJ/RJ, Brazil
First page: 168
Last page: 169

P-04. Pregnancy outcome after Day 3 embryo transfer versus blastocyst stage transfer in patients under 35 years

Bilibio João Paolo 1 2
Barros Jessica 2
Saba Junior Juscelino 2
Nascimento Fabio 1
Silva Bruno B da 1
Maciel Yasmin 2
Lorenzzoni Panila L 1
Afonso Jenny S L 2
Meireles Arivaldo 1
1 Clínica PRONATUS - Medicina Reprodutiva, Belém/ PA, Brazil
2 UFPA - Universidade Federal do Pará, Brazil
First page: 169
Last page: 169

P-05. Effect of endometrioma cysts on embryo quality and fertilization rate after IVF/ICSI

Lima Yanna A R de 1
Approbato Fabiana C 1
Maia Mônica C S 1
Ramos Marisa S 1
Brito Maria Z P 1
Santos Elisete N 1
Silveira Iulla A da 1
Approbato Mário S 1
1 Laboratório de Reprodução Humana, Hospital das Clínicas, Universidade Federal de Goiás/GO, Brazil
First page: 169
Last page: 169

P-06. Evaluation of oocyte profile of patients undergoing controlled ovarian stimulation (COS) with Elonva®

Neto José A L 1
Peixoto Ana P 1
Schuffner Alessandro 1
Rosa Vinicius B da 1
1 Clínica Conceber - Centro de Medicina Reprodutiva, Curitiba/ PR, Brazil
First page: 170
Last page: 170

P-07. Serological evaluation of Zika virus, in epidemic area, in asymptomatic infertility patients

Brandão Karina S A G 1
Oliveira Sofia A de 1
Viana Gersia A 1
Lessa Manoela C L 1
Muragaki Valentina N C 1
Trigo Ana C M 1
Filho Pedro P B 1
Lopes Joaquim R C 1
1 CENAFERT - Centro de Medicina Reprodutiva, Salvador/BA, Brazil
First page: 170
Last page: 170

P-08.High Progesterone levels at the initiation of stimulation cycles with antagonists and clinical pregnancy: still a concern?

Panaino Tatiana R 1
Silva Joyce B da 1
Tamm Maria A 1
Lira Paloma 1
Arêas Patricia C F 1
Mancebo Ana C A 1
Souza Marcelo M de 1
Antunes Roberto A 1
Souza Maria Do Carmo B de 1
1 Fertipraxis Centro de Reprodução Humana, Rio de Janeiro/ RJ, Brazil
First page: 170
Last page: 170

P-09. Ovarian grafts 10 days after xenotransplantation: folliculogenesis and recovery of viable oocytes

Dias Marco T 1
Alves Thalys J M 1
Marinho Fernanda E L 1
Gonzaga Jhéssica L M 1
Campos-Júnior Paulo H A 1
1 Universidade Federal de São João del Rei/MG, Brazil
First page: 171
Last page: 171

P-10. Ovarian vitrification does not affect the estradiol secretion and blood perfusion in autografted mice

Alves Thalys J M
Dias Marco T
Barreto Filipe P
Pereira Luiza A A C
Gonzaga Jhéssica L M
Campos-Júnior Paulo H A
1 Universidade Federal de São João del Rei/MG, Brazil
First page: 171
Last page: 171

P-11. Differential profile of transcripts in eutopic endometrium of infertile women with endometriosis and controls during the implantation window

Broi Michele G da 1
1 Faculdade de Medicina de Ribeirão Preto, Universidade de São Paulo (FMRP-USP), Brazil
First page: 171
Last page: 172

P-12. Transfer of vitrified embryos after estrogen administration or in ovulatory cycles

Moraes Leonardo A M de 1
Aguiar Luciana P T de 1
Oliveira Gabriella G de 1
Silva Felipe D 1
Brescia Erika M 1
Caires Camila O S 1
1 Clinica Fertibaby, Belo Horizonte/ MG, Brazil
First page: 172
Last page: 172

P-13. Children of donated gametes: tell or not to tell?

Marchi Bruna R de 1
Fácio Cássio L 1
Paula Ligiane A M de 1
Previato Ligia F 1
Araujo Filho Edilberto 1
1 Centro de Reprodução Humana de São José do Preto (CRH-Rio Preto), São José do Rio Preto, SP, Brasil
First page: 172
Last page: 172

P-14. Cryopreservation of human semen in medium TEST-yolk buffer or synthetic medium supplemented with phospholipid and antioxidant: Non-inferiority clinical trial

Silva Aline Bomfim 1
Sicchieri Fernanda 1
Vireque Alessandra A 2
Dantas Marilda H Y 1
Molina Carlos A F 1
Reis Rosana M dos 1
1 Faculdade de Medicina de Ribeirão Preto, Universidade de São Paulo (FMRP-USP)
2 Invitra Tecnologia da Reprodução Assistida
First page: 172
Last page: 172

P-15. Embryo re-biopsy for failure DNA amplification diagnosis by aCGH

Bueno Monique B 1
1 Igenomix - laboratório de medicina genética, Sao Paulo/ SP, Brazil
First page: 172
Last page: 173

P-16. The use of social networks to inform women on endometriosis and infertility: preliminary results

Carneiro Márcia M 1 2
Baroni Ana L L R 2
Candido Eduardo B 2
Silva Filho Agnaldo L da 2
Ferreira Márcia C F 1 2
1 Centro de Reprodução Humana MATER DEI Belo Horizonte-MG, Brasil
2 Departamento de Ginecologia e Obstetrícia Faculdade de Medicina da UFMG, Belo Horizonte-MG, Brasil
First page: 173
Last page: 173

P-17. Follicular flushing associated to oocyte retrieval in poor responders submitted to art increase the number of oocytes, embryos and pregnancy rates

Souza Anna L M 1
Sampaio Marcos 2
Geber Selmo 1 2
Noronha Graciele B 1
Coster Ludiana G R 1
Oliveira Roberta S G de 1
1 UFMG – Universidade Federal de Minas Gerais, Belo Horizonte/MG,  Brazil
2 ORIGEN – Center for Reproductive Medicine, Rio de Janeiro/RJ,  Brazil
First page: 173
Last page: 173

P-18. Preimplantation Genetic Diagnosis associated to Duchenne Muscular Dystrophy

Barbosa Caio P 1
Trevisan Camila M 1
Conceição Gabriel S 1
Santos Monise C 1
Christofolini Denise M 1
Bianco Bianca 1
1 Centro de Reprodução e Genética - Faculdade de Medicina do ABC
First page: 173
Last page: 174

P-19. Ovarian tissue bovine cryopreservation associated with in vitro folicular maturation in three-dimensional system to fertility preservation purposes

Bulgarelli Daiane L. 1
Campos Jacira R. 2
Gervázio Catieli G. 1
Batista Luciene A. 1
Higa Thais 1
Machado Marina M. 1
Silva Ana Carolina J. S. Rosa e 1
1 Gynecologic and Obstetrics Department, School of Medicine of Ribeirão Preto, University of São Paulo
2 Clínic Genics Medicina Reprodutiva e Genômica
First page: 174
Last page: 174

P-20. Blastocyst morphology affects the implantation rate in euploid embryo transfers

Kulmann Marcos I R 1
Hoher Marcos 1
Basso Carla G 1
Oliveira Norma P de 1
Frantz Gerta N 1
Dutra Caroline G 1
Bos-Mikich Adriana 1
Frantz Nilo 1
1 Centro de Reprodução Humana Nilo Frantz - Porto Alegre/RS, Brasil
First page: 174
Last page: 174

P-21. The chemokine receptor-2 (CCR2) and its ligand, CCL2 is expressed in oocytes and oviduct epithelium, respectively and can be related to oocyte transport

Santos Alana G A 1
Pereira Luiza A A C 1
Ciriaco Natália L C 1
Russo Remo C 2
Campos-Junior Paulo H A 1
1 Laboratório de Morfofisiologia e Biotecnologia Gonadal  - Universidade Federal de São João del Rei/MG, Brazil
2 Laboratório de Imunologia e Mecânica Pulmonar - Universidade Federal de Minas Gerais, Brazil
First page: 174
Last page: 175

P-22. Seminal parameters after different standard methods of sperm selection: comparisons between two techniques

Lunardi Franciele O 1
Pinheiro Neto João E 1
Serio Lilian M C 1
Alves Benner G 2
Alves Kele A 2
Cardoso Maria C 3
Aguiar Luciana P T de 4
Moraes Leonardo A M de 4
Paula Daniel P D de 1
1 Fertibaby Ceará, Fortaleza/CE, Brazil
2 Universidade Estadual do Ceará, Brazil
3 Centro de Fertilidade Vida, Rio de Janeiro/RJ, Brazil
4 Fertibaby Medicina Reprodutiva, Belo Horizonte/MG, Brazil
First page: 175
Last page: 175

P-23. Endometrial Receptivity Analysis for implantation failure in IVF: findings and results

Araujo Filho Edilberto 1
Fácio Cássio L 1
Paula Ligiane A M de 1
Previato Ligia F 1
1 Centro de Reprodução Humana de São José do Preto (CRH-Rio Preto), São José do Rio Preto/SP, Brazil
First page: 175
Last page: 176

P-24. Influence on the development of vitrified isolated secondary follicles after in vitro cultured in α-MEM and TCM base media in alginate matrix

Bulgarelli Daiane L. 1
Campos Jacira R. 2
Gervázio Catieli G. 1
Batista Luciene A. 1
Higa Thais T. 1
Zelinski Mary B. 3
Silva Ana Carolina J.S. Rosa e 1
1 Gynecologic and Obstetrics Department, School of Medicine of Ribeirão Preto, University of São Paulo
2 Clínic Genics Medicina Reprodutiva e Genômica.
3 Division of Reproductive & Developmental Sciences, Oregon National Primate Research Center, Oregon Health & Sciences University, Beaverton, Oregon, USA
First page: 176
Last page: 176

P-25. Predictors for oocyte recovery

de Conto Emily 1 2
Brandão Frederico A Reis 1
Genro Vanessa K 2
Chapon Rita de Cassia B 1 2
Silva Daniela S da 2
Cunha-Filho Joao S da 1 2
1 UFRGS - Universidade Federal do Rio Grande do Sul,  Porto Alegre/RS, Brazil
2 Insemine - Centro de Reprodução Humana, Porto Alegre/ RS, Brazil
First page: 176
Last page: 176

P-26. Effect of varicocelectomy on sperm DNA fragmentation, global DNA methylation, semen quality and hormonal parameters in man with varicocele

Santana Viviane P 1
Miranda-Furtado Cristiana L 1
Vasconcelos Maria A C 1
Dantas Marilda H Y 1
Molina Carlos A F 2
Reis Rosana M 1
1 Department of Gynecology and Obstetrics, Ribeirao Preto Medical School, University of Sao Paulo, Ribeirao Preto/SP, Brazil
2 Department of Surgery and Anatomy, Ribeirao Preto Medical School, University of Sao Paulo, Ribeirao Preto/SP, Brazil
First page: 176
Last page: 177

P-27. The embryo flash position immediately after IFV cycles transfer and assisted reproduction results

Brandão Karina S A G 1
Maciel Janaina J M 1
Leão Tassia S 1
Oliveira Sofia A de 1
Lopes Joaquim R C 1
1 CENAFERT - Centro de Medicina Reprodutiva, Salvador/ BA, Brazil
First page: 177
Last page: 177

P-28. Auto report of regularity of patient’s menstrual cycles. Medical history is very reliable to predict ovulation. A cross-sectional study

Sasaki Reinaldo S A 1
Approbato Mário S 1
Maia Mônica C S 1
Fleury Eliamar A B 1 2
Giviziez Christiane R 1
Zanluchi Neuma 3
1 Human Reproduction Center- Department of Obstetrics and Gynecology Clinical Hospital - Goias Federal University – Goiânia – Goiás – Brazil
2 Musical School and Scenic Arts/Goias Federal University
3 Hospital de Doenças Tropicais
First page: 177
Last page: 177

P-29. Aneuploidy rates in embryos biopsied on days 5 or 6

Tagliani-Ribeiro Alice 1
Azambuja Ricardo 1
1 Fertilitat - Centro de Medicina Reprodutiva, Porto Alegre/RS, Brasil
First page: 177
Last page: 177

P-30. Evaluation of pregnancy rate using two soft embryo transfer catheters

Polisseni Juliana 1
Assunção Carolina M de 1
Polisseni Fernanda 1
Rosa Josélio V 1
Oliveira Jociane V S 1
Coutinho Larissa M 1
Caetano João P J 1
1 Pró-Criar Medicina Reprodutiva, MG, Brazil
First page: 177
Last page: 178

P-31. Ovarian tissue vitrification in prepubertal wistar rats: comparative analysis among different protocols

Wietcovsky Leticia 1
Siewert Angélica 1
Lamim Tamara 1
Til David 1
Salvador Rafael A 1
Amaral Nicole L L 2
Frajblat Marcel 3
Amaral Vera L L 1
1 UNIVALI - Universidade do Vale do Itajaí, Itajai, SC, Brazil
2 CCV - Centro Clínico Veterinário, Itajai/ SC, Brazil
3 Universidade Federal do Rio de Janeiro – UFRJ, RJ, Brazil
First page: 178
Last page: 178

P-32. Vitrification of immature testicular tissue in wistar rats

Benvenutti Larissa 1
Wietcovsky Leticia 1
Velazquez Eduardo S A 1
Salvador Rafael A 1
Til David 1
Amaral Vera L L 1
1 UNIVALI - Universidade do Vale do Itajaí
First page: 178
Last page: 178

P-33. First pregnancy in Brazil resulting from criopreservated blastocyst obtained from in vitro matured oocytes

Scalco Priscila P C 1
Hoher Marcos 1
Frantz Nilo 1
Kulmann Marcos I R 1
Mattiello Simone S 1
Frantz Gerta 1
Oliveira Norma P de 1
Dutra Caroline G 1
Paguiarin Daiane M 1
1 Centro de Reprodução Humana Nilo Frantz, Porto Alegre/RS, Brazil
First page: 178
Last page: 179

P-34. Oocyte and embryo quality in endometriosis

Ribeiro Kazue H 1
Cerqueira Mila H R 1
Silva Gustavo C E 1
Perez Vanessa N P 1
Lima Rayssa A de 1
1 CLINIFERT – Florianópolis/SC, Brazil
First page: 179
Last page: 179

P-35. Does the blastocoele expansion degree interfere with blastocysts vitrification protocol?

Lamim Tamara 1
Amaral Vera L L 2
Til David 2
Salvador Rafael A 2
Frajblat Marcel 3
Siewert Angélica 1
Wietcovsky Leticia 1
1 Laboratório de Biotecnologia da Reprodução - LBR/UNIVALI, Itajaí/SC, Brazil
2 Universidade do Vale do Itajaí – UNIVALI,Itajaí/SC, Brazil
3 Universidade Federal do Rio de Janeiro – UFRJ/RJ, Brazil
First page: 179
Last page: 179

P-36. Prevalence of hysteroscopy alterations in women undergoing in vitro fertilization: cost-benefit analysis of screening hysteroscopy

Monteiro Cecília de Souza 1
1 Laboratório de Reprodução Humana do Hospital das Clínicas da Universidade Federal de Minas Gerais
First page: 179
Last page: 180

P-37. DNA fragmentation index can be reduced by dietary changes and antioxidant therapy increasing the euploidia rate in embryos undergoing preimplantation genetic screening: a case report

Kulmann Marcos I R 1
Silvestrim Rosa L 1
Oliveira Norma P de 1
Basso Carla G 1
Frantz Gerta N 1
Dutra Caroline G 1
Frantz Nilo 1
1 Centro de Reprodução Humana Nilo Frantz, Porto Alegre/RS, Brazil
First page: 180
Last page: 180

P-38. Endometriosis affects blastulation in art?

Conto Emily de 1 2
Silveira Juliana G 1
Genro Vanessa K 2
Chapon Rita de Cassia B 1 2
Silva Daniela S da 2
Cunha-Filho Joao S da 1 2
1 UFRGS - Universidade Federal do Rio Grande do Sul,  Porto Alegre/RS, Brazil
2 Insemine - Centro de Reprodução Humana, Porto Alegre, RS, Brazil
First page: 180
Last page: 180

P-39. Case report of zika virus during the controlled ovarian hyperstimulation: results from follicular fluid and cumulus cells

Araujo Filho Edilberto 1
Fácio Cássio L 1
Paula Ligiane A M de 1
Martinhago Ciro D 2
Previato Ligia F 1
1 Centro de Reprodução Humana de São José do Rio Preto (CRH-Rio Preto), São José do Rio Preto/ SP, Brazil
2 Chromosome Medicina Genômica, São Paulo/ SP, Brazil
First page: 180
Last page: 181

P-40. Oocyte per antral follicle count recuperation rate by using GnRH agonist to trigger follicle maturation in oocyte donation cycles

Aquino Ana P 1
Bonetti Tatiana C S 1
Motta Eduardo L A da 1
Serafini Paulo C 1
Devecchi Talita G S 1
Kimati Carolina 1
Barros Bruna 1
Domingues Thais S 1
1 Huntington Medicina Reprodutiva, Sao Paulo/SP, Brazil
First page: 181
Last page: 181

P-41. Learning about blastocyst culture from a pre-implantation genetic screening (PGS) program

Cardoso Maria C 1
Evangelista Alessandra 1
Cuzzi Juliana F. 2
Sartório Cássio A 1
Sant’anna Vivian 1
Werneck Caio L V 1
Aguiar Ana P S 1
Sá Paulo G de 1
Martins Maria C E C 1
1 Vida Centro de Fertilidade, Rio de Janeiro/RJ, Brazil
2 Genesis Genetics Brazil
First page: 181
Last page: 181

P-42. Two successful births using in vitro maturation (IVM) technique in same patient within a period of seven years

Dutra Caroline G 1
Mattiello Simone S 1
Frantz Gerta N 1
Oliveira Norma P de 1
Basso Carla G 1
Kulmann Marcos I R 1
Hoher Marcos 1
Frantz Nilo 1
1 Centro de Reprodução Humana Nilo Frantz - Porto Alegre/RS, Brazil
First page: 181
Last page: 182

P-43. The effect of body mass index on the assisted reproductive outcomes for polycystic ovary patients

Kulmann Marcos I R 1
Basso Carla G 1
Oliveira Norma P de 1
Frantz Gerta N 1
Dutra Caroline G 1
Chagas Joana C 1
Robin Fernanda 1
Frantz Nilo 1
1 Centro de Reprodução Humana Nilo Frantz,  Porto Alegre/ RS,  Brazil
First page: 182
Last page: 182

P-44. A molecular biology laboratory experience with preimplantation genetic screening (PGS) by the next generation sequencing (NGS)

Rosa Vinicius B da 1
Peixoto Ana P 1
Lucca Neto José A 1
Schuffner Alessandro 1
1 Clínica Conceber - Centro de Medicina Reprodutiva, Curitiba/PR, Brazil
First page: 182
Last page: 182

Freeze-all embryos policy in IVF/ICSI cycles. Chance of pregnancy after vitrified and thawed embryo transfer in a closed system, using Lupron® or Ovidrel® during the final maturation

Florencio Rodopiano S 1
Castro Lidia R Z O 1
Rocha Jane P 1
Batista Mariana P 1
Oliveira Vinicius A de 1
Castro Eduardo C 1
Rocha Mylena N C 1
1 Humana Medicina Reprodutiva Goiânia/ GO, Brazil
First page: 182
Last page: 183

P-46. Oocyte in vitro maturation technique in an oncofertility perspective

Lopes Juliana A 1
Jianini Bruna T G M 1
Rodrigues Jhenifer K. 1 2 3
1 Associação Instituto Sapientiae, São Paulo/SP, Brazil
2 In Vitro Consultoria, Belo Horizonte/MG, Brazil
3 Latin America Global Oncofertility Network Oncofertility Consortium
First page: 183
Last page: 183

P-47. Positive outcome of chance in ar cycles per season

Conto Emily de 1 2
Bohn Renata 1
Fleuri Marco Vinicius A S B 1
Genro Vanessa K 2
Chapon Rita de Cassia B 1 2
Silva Daniela S da 2
Cunha-Filho Joao S da 1 2
1 UFRGS - Universidade Federal do Rio Grande do Sul,  Porto Alegre/RS, Brazil
2 Insemine - Centro de Reprodução Humana, Porto Alegre/RS, Brazil
First page: 183
Last page: 183

P-48. IVF/ICSI Endometrial thickness on the day of final follicular maturation and chance of pregnancy in hyperstimulated cycles

Rocha Jane P 1
Finotti Marta C C F 1
Batista Mariana P 1
Borges Mirian R 1
Oliveira Vinicius A de 1
Castro Eduardo C 1
Florencio Rodopiano S 1
1 Humana Medicina Reprodutiva Goiânia/GO, Brazil
First page: 183
Last page: 184

P-49. Murine oocyte in vitro maturation, with and without ovarian stimulation, in different commercial media of embryo culture

Velazquez Eduardo S A 1
Til David 1
Salvador Rafael A 1
Frajblat Marcel 2
Amaral Vera L L 1
1 Universidade do Vale do Itajai-UNIVALI, Itajai/ SC, Brazil
2 Universidade Federal do Rio de Janeiro –UFRJ/ RJ, Brazil
First page: 184
Last page: 184

P-50. The nursing consultation in Assisted Human Reproduction

Aderaldo Janaina F 1
Prado Nanete C da Costa 2
Menezes Dayane J C de 2
Morais Kleber M 1 2
Nóbrega Edualeide J P B da 2
Torres Mychelle M G 2
Barbosa Kelley R N 1
1 Empresa Brasileira de Serviços Hospitalares – EBSERH, Brazil
2 Universidade Federal do Rio Grande do Norte – UFRN/RN, Brazil
First page: 184
Last page: 184

P-51. Xanthan gum as a cryoprotectant additive in human sperm preservation

Costa Sabrina M R J 1 2
Valverde Fernanda G 1
Souza Neto Conrado M 1
Silveira Erika C 2
Silveira George H C 2
Padilha Francine F 1
Verde Isabel B L 1
1 UNIT - Universidade Tiradentes, Aracaju/ SE, Brazil
2 CEMISE - Centro de Medicina Integrada de Sergipe, Aracaju/ SE, Brazil
First page: 185
Last page: 185

P-52. Clomiphene Citrate to prevent follicular rupture in oocytes and embryos vitrification cycles

Lopes Vinicius M 1
Brasileiro Jean P B 1
Lopes Joaquim R C 2
Tierno Natalia I Z 1
Lacerda Thaisa S 1
1 Instituto Verhum, Brasilia/DF, Brazil
2 CENAFERT - Centro de Medicina Reprodutiva, Salvador/BA, Brazil
First page: 185
Last page: 185

P-53. Genetic polymorphisms association with poor responder in patients undergoing in vitro fertilization

Meireles Arivaldo 1
Cunha-Filho Joao S da 2
Sales Pablo 1
Magalhaes Izabel 3
Nascimento Fabio 1
Lorenzzoni Panila L 1
Castro Jordana 3
Gama Thiago B 3
Bilibio João Paolo 1 3
1 Clínica PRONATUS - Medicina Reprodutiva, Belém/ PA, Brazil
2 UFRGS - Universidade Federal do Rio Grande do Sul,  Porto Alegre/ RS, Brazil
3 UFPA - Universidade Federal do Pará, Brazil
First page: 185
Last page: 186

P-54. Fresh embryo transfer in IVF/ICSI cycles using Lupron® or Ovidrel® for final maturation and chance of pregnancy

Oliveira Vinicius A de 1
Borges Mirian R 1
Florencio Rodopiano S 1
Rocha Jane P 1
Rocha Mylena N C 1
Castro Corival L A de 1
Santos Fernando C 1
1 Humana Medicina Reprodutiva Goiânia/ GO, Brazil
First page: 186
Last page: 186

P-55. Fertility preservation for men: review on recent developed techniques for cryopreservation of a small number of sperm cells

Dutra Josiane B 1
Jianini Bruna T G M 1
Rodrigues Jhenifer K
1
2
3
1 Associação Instituto Sapientiae, São Paulo/ SP, Brazil
2 In Vitro Consultoria, Belo Horizonte/ MG, Brazil
3 Latin America Global Oncofertility Network Oncofertility Consortium
First page: 186
Last page: 186

P-56. Pre-implantation genetic screening (PGS) and the influence of maternal age on the incidence of embryonic aneuploidy

Pedrosa Moísa L 1
Martins Bruna O 1
Aguiar Luiza M S 1
Carneiro Márcia M 1
Cota Ana Márcia M 1
Ferreira Márcia C F 1
Lamaita Rívia M 1
1 Reprodução Humana Mater Dei, Belo Horizonte/MG, Brazil
First page: 186
Last page: 187

P-57. Germinal tissue cryopreservation: potential fertility preservation alternative for male and female cancer patients

Reis Mariana A S 1
Rodrigues Jhenifer K 1 2 3
1 Associação Instituto Sapientiae, São Paulo/SP, Brazil
2 In Vitro Consultoria, Belo Horizonte/MG, Brazil
3 Latin America Global Oncofertility Network - Oncofertility Consortium
First page: 187
Last page: 187

P-58. Age assessment, type of infertility, marital union time and patient stress symptoms of infertile women submitted to music therapy in a brazilian university center

Fleury Eliamar A B 1 2
Approbato Mário S 1
Sasaki Reinaldo S A 1
Maia Mônica C S 1
Ramos Marisa S 1
Approbato Fabiana C 1
Magri Maíra R 3
Oliveira Flávia N M de 4
Lima Yanna A R de 1
1 Human Reproduction Center - Department of Obstetrics and Gynecology Clinical Hospital - Goias Federal University – Brazil
2 Musical School and Scenic Arts/Goiás Federal University
3 Catholic University of Goiás – Brazil
4 Health Secretariat of District Federal
First page: 187
Last page: 187

P-59.The use of 1 (one) frozen donor semen straw in 2 (two) fertilization in vitro cycles

Silva Beatriz M 1
Costa Rosaly R 1
Sousa Victor E T de 1
Macedo Yamara A 1
1 Centro de Ensino e Pesquisa em Reprodução Assistida do Hospital Materno Infantil de Brasília - CEPRA/HMIB
First page: 187
Last page: 188

P-60. The reproductive profile of patients undergoing genetic analysis

Barbosa Caio P 1 2
Santos Monise C 1 2
Trevisan Camila M 1 2
Bianco Bianca 1 2
Christofolini Denise M 1 2
1 Instituto Ideia Fértil
2 Faculdade de Medicina do ABC
First page: 188
Last page: 188

P-61. The impact of immature oocytes quantity on the rates of fertilization, cleavage, embryo development and total pregnancy in ICSI cycles

Polisseni Juliana 1
Assunção Carolina M de 1
Polisseni Fernanda 1
Rosa Josélio V 1
Oliveira Jociane V S 1
Coutinho Larissa M 1
Caetano João P J 1
1 Pró-Criar Medicina Reprodutiva, MG, Brazil
First page: 188
Last page: 188

P-62. Use of subcutaneous granulocyte colony stimulating factor (Filgrastine®) in ICSI procedure and fresh embryo transfer, for patients with reference to the age group less than or equal to 40 years old, regardless of endometrial thickness measure

Florencio Rodopiano S 1
Rocha Mylena N C 1
Castro Corival L A de 1
Santos Fernando C 1
Finotti Marta C C F 1
Castro Eduardo C 1
Oliveira Vinicius A de 1
1 Humana Medicina Reprodutiva Goiânia/ GO, Brazil
First page: 188
Last page: 189

P-63. Ethics involved on fertility preservation for pediatric cancer patients: review and discussion

Inácio Irislande 1
Branco Fernando Castelo 1
De Carvalho Bruno Ramalho 3
Rodrigues Jhenifer Kliemchen 1 2 3
1 Associação Instituto Sapientiae, São Paulo/ SP, Brazil
2 In Vitro Consultoria, Belo Horizonte/ MG, Brazil
3 Latin America Oncofertility Network - Oncofertility Consortium
First page: 189
Last page: 189

P-64. The use of erotic visual stimuli during semen collection according to the purpose of the sample

Correa Beatriz M Silva 1
De Araújo Eduardo S 1
Correa Frederico J S 1
Chaussard Isadora S 1
1 FERTIL CARE - Centro de Reprodução Humana, Brasília/DF, Brazil
First page: 189
Last page: 190

P-65. Evaluation of assisted reproduction services in the city of Ribeirão Preto/SP

Sanches Wellington 1
Nogueira Fabiana P N 1
Dias Margareth J 2
1 Divisão de Vigilância Sanitária da Secretaria Municipal da Saúde de Ribeirão Preto/ SP, Brazil
2 Grupo Técnico Estadual de Vigilância Sanitária de Ribeirão Preto/ SP, Brazil
First page: 190
Last page: 190

P-66. Supraphysiological concentrations of gonadal hormones reduce the anxiety behavior in female rats submitted to psychogenic stress

Sueur-Maluf Luciana Le 1
Mariotti Flora F N 1
Pimpão Giovanna 1
Antunes Hanna K M 1
Melo Augusto A de 1
Baptista Heloísa A 1
Viana Milena B 1
Céspedes Isabel C 1
1 Universidade Federal de São Paulo – UNIFESP, SP, Brazil
First page: 190
Last page: 190

P-67. Equipment for concentration and comparative motility sperm

Navogino Regina A A 1
1 Setor de Analise Seminal Grupo Fleury Medicina e Saude, Brazil
First page: 190
Last page: 191

P-68. Adding the number of follicles 14-16mm to hCG trigger criteria makes a difference?

Cota Ana Márcia M 1
Ferreira Márcia C F 1
Pedrosa Moísa L 1
Martins Bruna O 1
Lamaita Rívia M 1
Carneiro Márcia M 1
1 Reprodução Humana Mater Dei, Belo Horizonte/MG, Brazil
First page: 191
Last page: 191

P-69. Psychological profile of pacients seeking tertiary care of infertility

Bartmann Ana K 1
Silva Lucas L M da 1
Melo Amélia G V de 1
Saran Paulo C 1
Rezek Uebe C 1
1 Universidade de Ribeirão Preto - Clínica Ana Bartmann
First page: 191
Last page: 191

P-70. Implementing the nursing service in a new center for human reproduction

Dias Giselle S 1
Silva Beatriz M 1
Correa Frederico J S 1
1 FERTIL CARE - Centro de Reprodução Humana, Brasília/DF, Brazil
First page: 191
Last page: 191

P-71. Cognitive behavioral therapy: assisted human reproduction in women

Marchi Bruna R de 1
Fácio Cássio L 1
Paula Ligiane A M de 1
Previato Ligia F 1
Araujo Filho Edilberto 1
1 Centro de Reprodução Humana de São José do Preto (CRH-Rio Preto), São José do Rio Preto/ SP, Brazil
First page: 191
Last page: 192

P-72. A case report supporting the concept that some women have a predisposition for maternal meiosis erros

Polisseni Juliana 1
Assunção Carolina M de 1
Polisseni Fernanda 1
Rosa Josélio V 1
Oliveira Jociane V S 1
Coutinho Larissa M 1
Caetano João P J 1
1 Pró-Criar Medicina Reprodutiva, MG, Brazil
First page: 192
Last page: 192

P-73. A prospective study comparing two embryo transfer soft catheters

Conto Emily de 1 2
Schuster Artur K 1
Genro Vanessa K 2
Chapon Rita de Cassia B 1 2
Silva Daniela S da 1 2
Cunha-Filho Joao S da 1 2
1 UFRGS - Universidade Federal do Rio Grande do Sul,  Porto Alegre/ RS, Brazil
2 Insemine - Centro de Reprodução Humana, Porto Alegre/ RS, Brazil
First page: 192
Last page: 192

P-74. Embryo stage of development is not decisive for reproductive outcomes in frozen-thawed embryo transfer cycles

Carvalho Bruno R de 1
Barbosa Marina W P 1
Bonesi Helena 1
Sobrinho David B Gomes 1
Cabral Iris O 1
Barbosa Antônio C P 1
Silva Adelino A 1
Iglésias Jose R 1
Nakagawa Hitomi M 1
1 Genesis - Center for Assistance in Human Reproduction, Brasília/ DF, Brazil
First page: 192
Last page: 193

